# MiR-215-5p Reduces Liver Metastasis in an Experimental Model of Colorectal Cancer through Regulation of ECM-Receptor Interactions and Focal Adhesion

**DOI:** 10.3390/cancers12123518

**Published:** 2020-11-26

**Authors:** Tana Machackova, Petra Vychytilova-Faltejskova, Kamila Souckova, Karolina Trachtova, Dominika Brchnelova, Marek Svoboda, Igor Kiss, Vladimir Prochazka, Zdenek Kala, Ondrej Slaby

**Affiliations:** 1 Central European Institute of Technology, Masaryk University, 625 00 Brno, Czech Republic; tana.machackova@ceitec.muni.cz (T.M.); vychytilova.petra@seznam.cz (P.V.-F.); 11949@mail.muni.cz (K.S.); trachtova@mail.muni.cz (K.T.); d.brchnelova@gmail.com (D.B.); 2 Department of Comprehensive Cancer Care, Masaryk Memorial Cancer Institute, Faculty of Medicine, Masaryk University, 602 00 Brno, Czech Republic; msvoboda@mou.cz (M.S.); kiss@mou.cz (I.K.); 3 Department of Surgery, Faculty Hospital Brno and Faculty of Medicine, Masaryk University, 625 00 Brno, Czech Republic; Prochazka.Vladimir@fnbrno.cz (V.P.); Kala.Zdenek@fnbrno.cz (Z.K.); 4 Department of Biology, Faculty of Medicine, Masaryk University, 625 00 Brno, Czech Republic

**Keywords:** miR-215-5p, metastasis, colorectal cancer, focal adhesion, extracellular matrix-receptor interaction

## Abstract

**Simple Summary:**

Decreased expression of miR-215-5-p was found in tumor tissue of patients with colorectal cancer (CRC) in comparison to healthy colon tissue. Moreover, expression levels of miR-215-5p were further decreased in metastatic lesions compared to primary tumor tissue. Overall, CRC patients with lower expression of miR-215-5p in tumors had significantly shorter overall survival and a higher chance of metastasis. This study aimed to examine the effects of miR-215-5p supplementation on the metastatic potential of CRC. MiR-215-5p was found to decrease invasiveness, migratory capacity, tumorigenicity, and metastasis formation. Finally, transcriptome analysis identified signaling pathways involved in the process, and subsequent RT-qPCR validation indicates CTNNBIP1 to be a direct target of this microRNA. These results bring new insight into miR-215-5p biology, a molecule that could potentially serve as a promising target for CRC patients’ future therapeutic strategies.

**Abstract:**

**Background:** Growing evidence suggests that miR-215-5p is a tumor suppressor in colorectal cancer (CRC); however, its role in metastasis remains unclear. This study evaluates the effects of miR-215 overexpression on the metastatic potential of CRC. Methods: CRC cell lines were stably transfected with miR-215-5p and used for in vitro and in vivo functional analyses. Next-generation sequencing and RT-qPCR were performed to study changes on the mRNA level. **Results**: Overexpression of miR-215-5p significantly reduced the clonogenic potential, migration, and invasiveness of CRC cells in vitro and tumor weight and volume, and liver metastasis in vivo. Transcriptome analysis revealed mRNAs regulated by miR-215-5p and RT-qPCR confirmed results for seven selected genes. Significantly elevated levels of CTNNBIP1 were also observed in patients’ primary tumors and liver metastases compared to adjacent tissues, indicating its direct regulation by miR-215-5p. Gene Ontology and KEGG pathway analysis identified cellular processes and pathways associated with miR-215-5p deregulation. **Conclusions**: MiR-215-5p suppresses the metastatic potential of CRC cells through the regulation of divergent molecular pathways, including extracellular-matrix-receptor interaction and focal adhesion. Although the specific targets of miR-215-5p contributing to the formation of distant metastases must be further elucidated, this miRNA could serve as a promising target for CRC patients’ future therapeutic strategies.

## 1. Introduction

Colorectal cancer (CRC) has the third-highest incidence and is the second most common cancer-related death worldwide [1]. Despite the global adoption of screening programs, approximately 25% of CRC patients are diagnosed with metastatic disease [2,3]. The evolution of CRC can take years, sometimes decades, and the process was previously described in molecular detail [4]. CRC is a disease with a high level of heterogeneity on the molecular level and complex regulation. New molecules involved in the process are continuously discovered [5]. MicroRNAs (miRNAs) are a class of small non-coding RNAs that post-transcriptionally regulate gene expression by binding to 3′ untranslated regions of target mRNAs [6]. They have been shown to have important implications in cancer biology as they may act as tumor suppressors or oncogenes, depending on the localization and involvement in different cellular processes [7].

Recently, miR-215-5p has been found to be deregulated in many pathological conditions, including CRC. Nevertheless, its precise role in the pathogenesis of the disease remains unclear. Interestingly, there is conservation in its structure among different species indicating that this miRNA may have vital functions that are maintained during evolution [8]. Previously, miR-215-5p was detected in colon epithelial cells, colonic enteroids, and flow-isolated colon epithelium; however, its expression was not confirmed in fibroblasts and endothelial cells. Similarly, to protein-coding genes, miRNAs’ expression may be regulated on the transcriptional or post-transcriptional level. Several different molecules have been previously identified to affect the transcription of miR-215-5p, including p53 [9], caudal type homeobox 1 (CDX1) [10], HNF1/4α [11], or nuclear factor κB NF-κB) [12]. Importantly, over-expression of miR-215-5p may be induced by hypoxia [13], while various long non-coding RNAs (lncRNAs) may act as molecular sponges to regulate miR-215-5p expression negatively [14,15,16]. Previous studies described miR-215-5p involvement in numerous basic cellular processes, including cell and tissue development, cell survival, cell cycle and proliferation, cell migration and invasion, cellular microenvironment, and metabolism [17]. To this day, several research groups also investigated the clinical utility of miR-215-5p as a potential diagnostic, prognostic, and predictive biomarker in CRC. In 2008, Braun et al. [9] published the first study dealing with deregulated levels of miR-215-5p in CRC. Its expression was significantly downregulated in early-stage tumors of both genetic and inflammatory origin, indicating its involvement in the early stages of malignant transformation [18]. Low levels of miR-215-5p have been further associated with advanced clinical stage, undifferentiated tumors, lymph node positivity, presence of distant metastases [19], shorter overall survival (OS) [20], decreased response to 5-FU adjuvant chemotherapy, and high probability of 3-year relapse [21].

Previously, we have described significantly down-regulated levels of miR-215-5p not only in primary tumors of CRC patients but also in corresponding liver metastases [20]. Despite the growing number of studies analyzing the role of miR-215-5p in cancer pathogenesis and metastases formation, precise mechanisms are still not understood. Thus, we decided to use a stable transfection of miR-215-5p into three different CRC cell lines to study the effects of its supplementation on the metastatic process. In vitro functional analyses were used to examine the effects of miR-215-5p overexpression on CRC cells’ clonogenic potential and migratory and invasive capacity. Further, in vivo model was established to evaluate the effects of this miRNA on tumor growth and the formation of distant metastases. Moreover, next-generation sequencing (NGS) and transcriptome analysis were utilized to study changes on mRNA level induced by overexpression of miR-215-5p, with the aim to identify potential direct or indirect targets of this miRNA and further describe its involvement in various cellular processes and molecular pathways with the special focus on metastasis.

## 2. Results

### 2.1. Selection of Stably Transfected Clones

In our previous study, we described significant downregulation of miR-215-5p in primary CRC tumors and corresponding liver metastases [20]. Thus, we decided for stable transfection of miR-215-5p into three different CRC cell lines to further study the effects of its supplementation on the metastatic process. Stably transfected clones were selected according to the expression of miR-215-5p (clones with high levels were selected), the morphology of control cells was examined, and clones with similar morphology to original cell lines were used (Supplementary File 1).

### 2.2. MiR-215-5p Inhibits the Clonogenic Potential, Arrests the Cell Cycle, and Induce Apoptosis of CRC Cells

**Figure 1:**
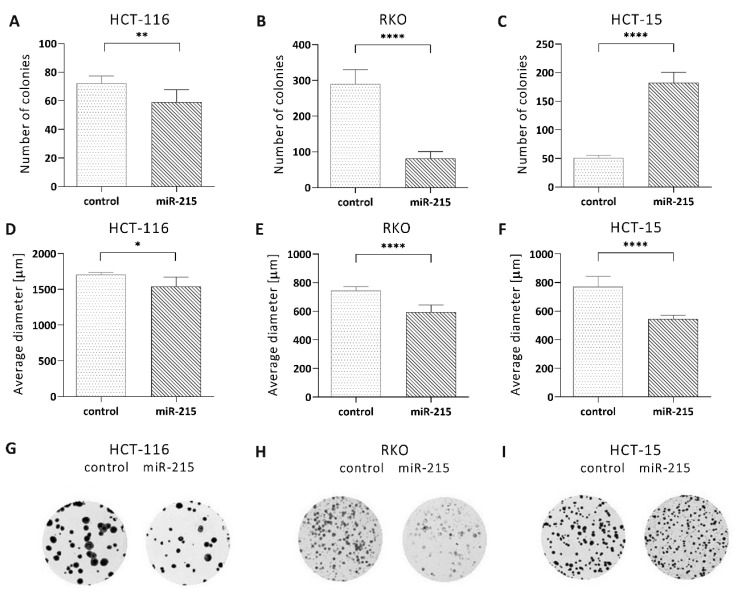
Colony formation assay: (**A**–**C**), number of colonies—overexpression of miR-215-5p significantly reduced the number of colonies in HCT-116 and RKO cells and increased the number of colonies in HCT-15 cells. (**D**–**F**), the colonies’ diameter—overexpression of miR-215-5p significantly reduced the diameter of colonies in the case of HCT-116, RKO, and HCT-15 cells. (**G**–**I**), representative pictures of colonies stained with crystal violet. * *p* < 0.05, ** *p* < 0.01, *** *p* < 0.001, **** *p* < 0.0001.

Clonogenic potential of individual clones with increased expression of miR-215-5p was significantly decreased in HCT-116 (*p* = 0.0090) (Figure 1A,G) and RKO cells (*p* < 0.0001) (Figure 1B,H). However, cell line HCT-15 showed the opposite results repeatedly, and overexpression of miR-215-5p led to significantly increased clonogenic potential of this cell line in vitro (*p* < 0.0001) (Figure 1C,I). Analysis of the size of formed colonies showed that overexpression of miR-215-5p resulted in a significantly smaller diameter of the colonies of HCT-116 (*p* = 0.0126) (Figure 1D), RKO (*p* < 0.0001) (Figure 1E), and HCT-15 (*p* < 0.0001) cells (Figure 1F). Flow cytometric analysis proved the arrest of cell cycle in G0/G1 phase in case of RKO cells (*p* < 0.0001) and in S phase in case of HCT-116 (*p* < 0.001) and HCT-15 (*p* < 0.05) cell lines (Supplementary File 2). Further, miR-215-5p overexpression led to the higher number of early apoptotic HCT-15 cells (*p* < 0.01) and late apoptosis/dead of HCT-116 (*p* < 0.001) and RKO *(p* < 0.01) cells was significantly increased (Supplementary File 2).

### 2.3. MiR-215-5p Inhibits Cell Migration/Invasion of HCT-15 Cells In Vitro

**Figure 2:**
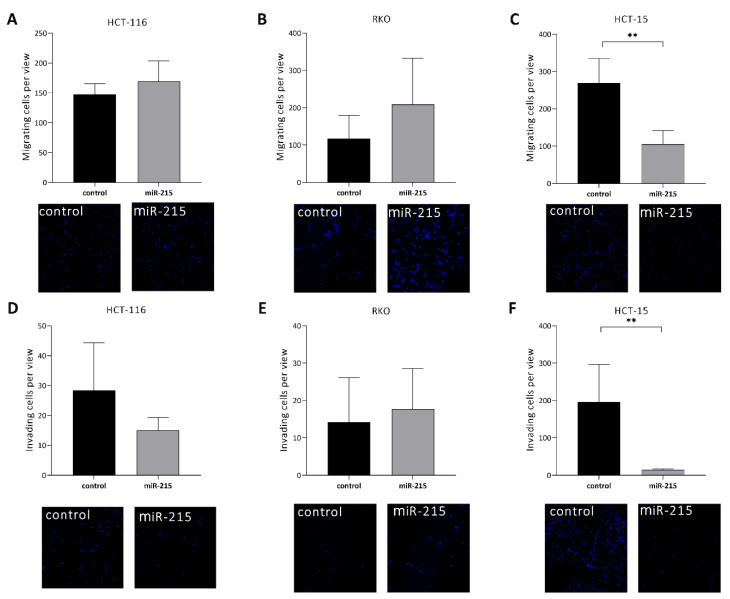
Transwell migration/invasion assay: (**A**–**C**) graphs of migrating cells per view, and representative pictures of migrated cells stained with Hoechst 33342. Overexpression of miR-215-5p significantly reduced the number of migrating HCT-15 cells. (**D**–**F**), and graphs of invading cells per view and representative pictures of invaded cells stained with Hoechst 33342. Overexpression of miR-215-5p significantly reduced the number of invading HCT-15 cells and visibly reduced the number of invading HCT-116 cells. * *p* < 0.05, ** *p* < 0.01, *** *p* < 0.001, **** *p* < 0.0001.

Transwell migration assay showed that HCT-15 cells with overexpression of miR-215-5p have significantly decreased migratory capacity (*p* = 0.0012) (Figure 2C). In contrast to that, HCT-116 (Figure 2A) and RKO cells (Figure 2B) with overexpression of miR-215-5p showed an opposite trend. Transwell invasion assay showed that overexpression of miR-215-5p led to significantly decreased invasive potential in the case of HCT-15 cells (*p* = 0.0037) (Figure 2F). HCT-116 cells showed a trend of decreased invasion of cells with miR-215-5p overexpression (Figure 2D). In contrast to that, the invasion of RKO cells showed the opposite trend (Figure 2E).

### 2.4. MiR-215-5p Inhibits CRC Cells Tumor Growth In Vivo

**Figure 3:**
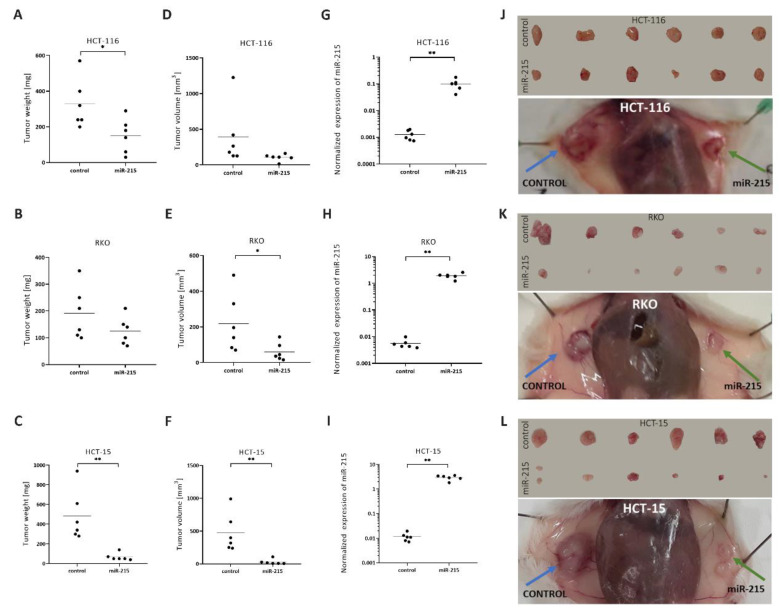
Tumorigenicity assay: (**A**–**C**), graphs of tumor weights—overexpression of miR-215-5p significantly reduced tumor weight in the case of HCT-116 and HCT-15 cells, and a similar trend was observed in the case of RKO cells. (**D**–**F**), graphs of tumor volumes—overexpression of miR-215-5p significantly reduced tumor volume in the case of RKO and HCT-15 cells, cell line HCT-116 showed a similar trend. (**G**–**I**), graphs of normalized (RNU48) expression of miR-215-5p in tumors. Expression of miR-215-5p remained significantly increased in HCT-116, RKO, and HCT-15 tumors. (**J**–**L**), pictures of removed control/miR-215-5p tumors and representative pictures of subcutaneous control/miR-215-5p tumors in the animal model. * *p* < 0.05, ** *p* < 0.01, *** *p* < 0.001, **** *p* < 0.0001.

Using NSG mice and tumorigenicity assay, we observed the tumor-suppressor effects of miR-215-5p. MiR-215-5p cell tumors had significantly lower weight than control cell tumors in case of HCT-116 (*p* = 0.0282) (Figure 3A) and HCT-15 (*p* = 0.0027) (Figure 3C). Moreover, in all three cell lines, miR-215-5p overexpression resulted in smaller tumors (Figure 3J–L). Tumor volume followed the trend and miR-215-5p overexpression significantly decreased tumor volume in RKO (*p* = 0.0464) and HCT-15 (*p* = 0.0042) miR-215 cells (Figure 3E,F). Expression analyses of miR-215-5p confirmed that its expression remains increased throughout the implantation and tumor growth, and miR-215-5p cell tumors showed significantly increased expression of miR-215-5p compared to control cell tumors in all three cell lines—HCT-116 (*p* = 0.0003), RKO (*p* < 0.0001), HCT-15 (*p* < 0.0001) (Figure 3G–I).

### 2.5. MiR-215-5p Inhibits the Metastatic Potential of CRC Cells In Vivo

**Figure 4:**
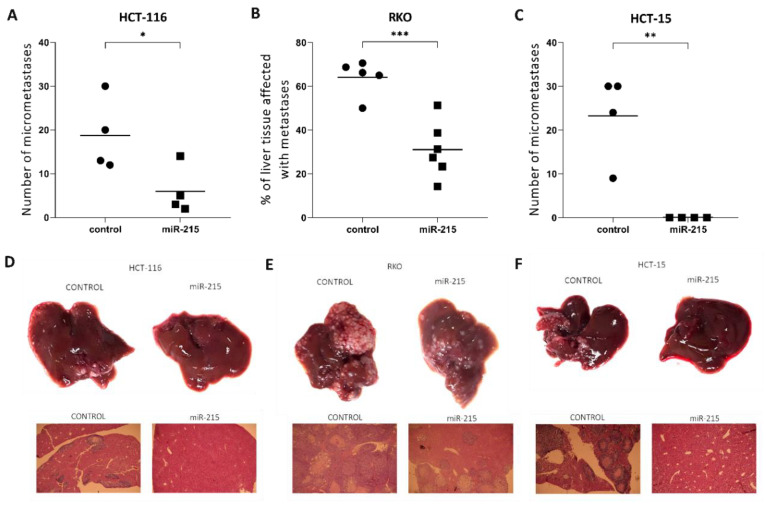
Intra-splenic metastatic model: (**A**–**C**), graphs with numbers of metastases—intra-splenic injection of cells with miR-215-5p overexpression resulted in a significantly smaller area affected by metastases/number of metastases in all three cell lines (**D**–**F**), representative micro/macro pictures of the liver with metastases. * *p* < 0.05, ** *p* < 0.01, *** *p* < 0.001, **** *p* < 0.0001.

The intra-splenic metastatic model using NSG mice showed the most consistent results of all experiments in this study. All three cell lines showed a similar trend of decreased metastatic potential of miR-215-5p cells in the liver. Statistically significant results were observed in case of HCT-116 (*p* = 0.0427), RKO (*p* = 0.0008), and HCT-15 (*p* = 0.0034) cell lines (Figure 4A–C).

### 2.6. Possible Molecular Mechanism of Metastatic Process Regulated by miR-215-5p via Involvement of ECM-Receptor Interactions and Focal Adhesion Pathways

**Figure 5:**
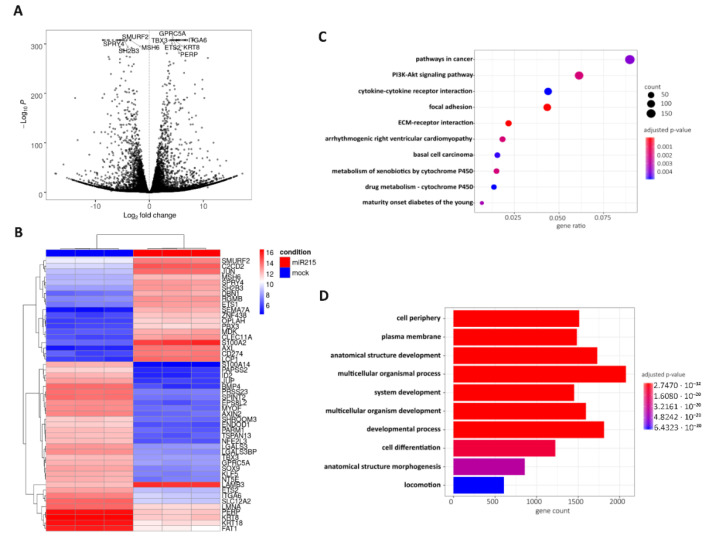
Transcriptome profiling: (**A**) Vulcano plot with the top ten most significantly deregulated mRNAs in HCT-15-miR-215-5p cells in comparison to control HCT-15 cells. (**B**) hierarchical clustrogram and heatmap of the top fifty deregulated mRNAs between HCT-15-miR-215-5p cells (red: miR-215-5p) and control HCT-15 cells (blue: mock). Genes clustered using unsupervised clustering. (**C**) KEGG pathway analysis—the top ten signaling pathways regulated by miR-215-5p in HCT-15 cells. (**D**) Gene Ontology overrepresentation analysis—top 10 overrepresented Gene Ontology terms (across all three sub-ontologies) regulated by miR-215-5p in HCT-15 cells.

**Figure 6:**
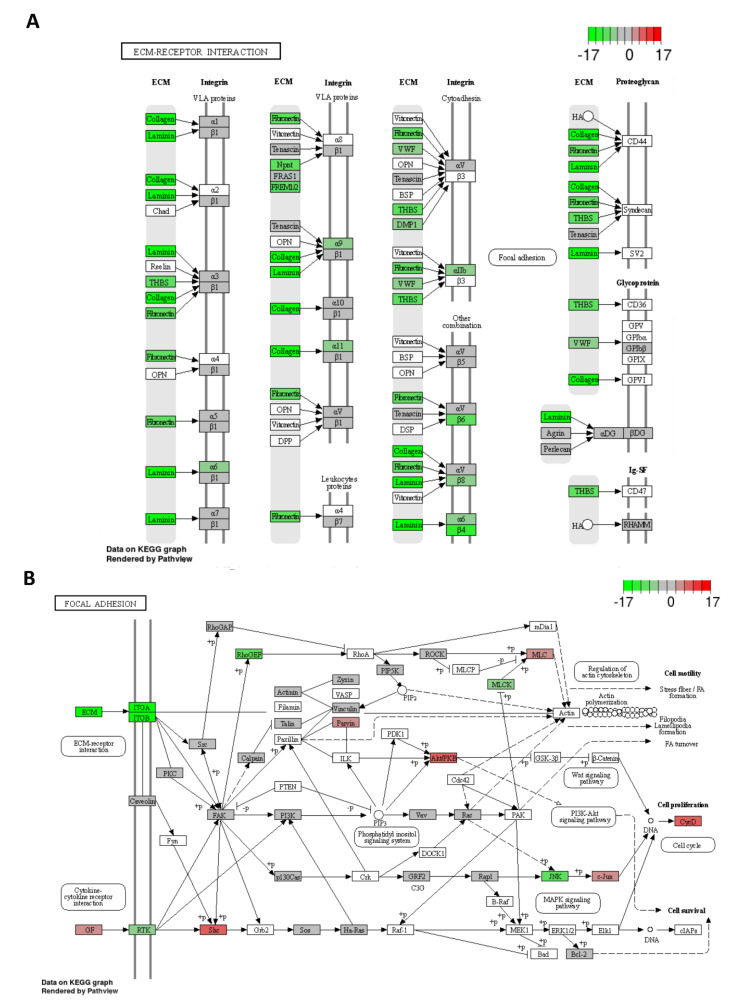
Top two signaling pathways regulated by miR-215-5p in CRC. (**A**) An Extra-cellular matrix-receptor interaction. (**B**) Focal adhesion pathway (Green—downregulated in miR-215-5p cells in comparison to control cells, Red—upregulated in miR-215-5p cells in comparison to control cells).

Sequencing data underwent differential expression analysis, and 8847 mRNAs with significantly (*p* adjust. < 0.001) deregulated expression in miR-215-5p transfected HCT-15 cells compared to HCT-15 control cells were identified (Figure 5A, Supplementary File 3). Among the top 50 deregulated mRNAs based on the adjusted p-value, 20 mRNAs were upregulated and 30 downregulated in miR-215-5p transfected cells (Figure 5B). KEGG pathway analysis proved three of these genes to be associated with focal adhesion (JUN, ITGA6, LAMB3), while CD274 is an important cell adhesion molecule. In addition, several genes associated with signaling pathways regulating pluripotency of stem cells (BMP4, AXIN2, ID2, TBX3) or Wnt signaling (AXIN2) have been identified as deregulated after miR-215-5p transfection. Subsequently, KEGG pathway analysis was also employed to identify signaling pathways possibly regulated by miR-215-5p in CRC. In total, miR-215-5p was connected with statistical significance (*p* adjust. < 0.05) to 27 different pathways. Top 10 deregulated pathways are shown in Figure 5C and Table 1. Subsequently, only pathways associated with metastatic processes have been further analyzed. In total, 273 mRNAs involved in 12 different pathways have been found to be significantly downregulated in response to miR-215-5p transfection (*p* < 0.05; at least 100 raw reads in all three samples from one condition—miR-215-5p or mock—in average; Supplementary File 4) including focal adhesion (66 mRNAs; Supplementary File 5), extracellular matrix-receptor interaction (30 mRNAs; Supplementary File 6), adherent junctions (26 mRNAs), tight junctions (52 mRNAs), gap junctions (21 mRNAs), Wnt signaling pathway (44 mRNAs), cell adhesion molecules (29 mRNAs), proteoglycans with the function in cancer (67 mRNAs), signaling pathways regulating pluripotency of stem cells (39 mRNAs), HIF-1 signaling pathway (33 mRNAs), and VEGF-signaling pathway (22 mRNAs). From those, seven mRNAs (Moesin (MSN), cAMP responsive element binding protein 5 (CREB5), catenin beta interacting protein 1 (CTNNBIP1), Protein kinase cAMP-activated catalytic subunit beta (PRKACB), Myosin Light Chain Kinase (MYLK), A receptor type 2B (ACVR2A), and Cbl proto-oncogene (CBL)) are predicted to be potential direct targets of miR-215-5p based on the TargetScan 7.2 database, while the others are probably the indirect targets of this miRNA. Finally, Gene Ontology (GO) overrepresentation analysis was used to investigate the association of supplementation with miR-215-5p with GO terms in three sub-ontologies (cellular components, molecular functions, biological processes). Top 10 GO terms for each sub-ontology are listed in Table 2. From those, cell periphery, plasma membrane, and locomotion (Figure 5D) indicate the connection between miR-215-5p expression and KEGG extra-cellular matrix (ECM)-interaction (Figure 6A) and focal adhesion (Figure 6B) pathways. Altogether, these data indicate highly probable involvement of miR-215-5p in the metastatic processes of CRC.

### 2.7. Validation of Potential miR-215-5p Targets

**Figure 7:**
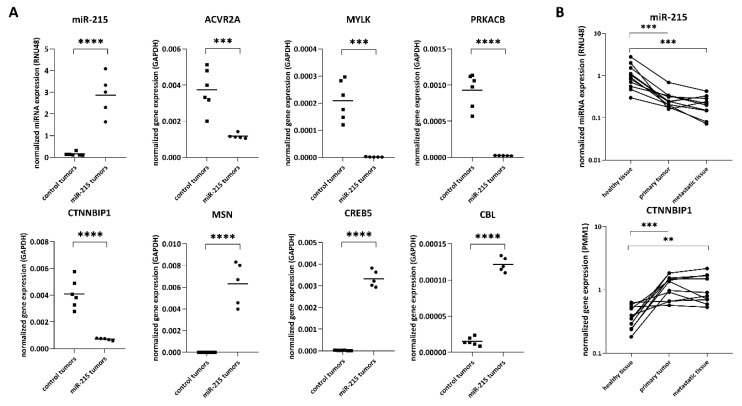
Validation of sequencing data by RT-qPCR. (**A**) Expression analyses of subcutaneous tumors generated in NSG mice using HCT-15 cells stably transfected with miR-215-5p and control cells. (**B**) Expression analyses of healthy colon tissue, primary tumor, and metastatic liver tissue of CRC patients. * *p* < 0.05, ** *p* < 0.01, *** *p* < 0.001, **** *p* < 0.0001.

Targetscan 7.2 database identified seven mRNAs (MSN, CREB5, CTNNBIP1, PRKACB, MYLK, ACVR2A, and CBL) as potential direct targets of miR-215-5p based on a comparison of sequencing data of HCT-15 cells stably transfected with miR-215 and control cells (only genes with *p* < 0.05 and at least 100 raw reads in all three samples from one condition have been analyzed). Validation of sequencing results by RT-qPCR confirmed the elevated levels of miR-215-5p (*p* < 0.0001) in tumors with its stable expression compared to control tumors as well as deregulation of all seven genes in subcutaneous tumors generated in NSG mice from HCT-15 cells (Figure 7A). While the expression of ACVR2A (*p* = 0.0008), MYLK (*p* = 0.0001), PRKACB (*p* < 0.0001), and CTNNBIP1 (*p* < 0.0001) was significantly downregulated, elevated levels of MSN (*p* < 0.0001), CREB5 (*p* < 0.0001), and CBL (*p* < 0.0001) were detected in tumors overexpressing miR-215-5p compared to tumors from control cells. These data are in accordance with the results of NGS. Subsequently, expression analysis of CRC patient samples proved significantly lower levels of miR-215-5p in primary tumor tissue (*p* = 0.001) as well as in paired metastatic tissue (*p* = 0.001) compared to adjacent colon tissue (Figure 7B). Concerning the potential target genes of this miRNA, only the expression of CTNNBIP1 was significantly upregulated in both, primary (*p* = 0.001) and liver metastatic (*p* = 0.0098) tumor tissue of CRC patients compared to adjacent colon tissue (Figure 7B).

## 3. Discussion

MiR-215-5p has been repeatedly shown to have a role in the regulation of the pathogenesis of various cancers. Tumor suppressor effects of this miRNA were observed in the case of non-small cell lung cancer via direct targeting of zinc finger E-box binding homeobox 2 (ZEB2) and inhibition of the epithelial-mesenchymal transition (EMT) process [22] and by downregulation of matrix metalloproteinase-16 (MMP16) expression [23]. Similarly, miR-215-5p was reported to act as a tumor suppressor in breast cancer by targeting Sox9 [24] and AKT serine/threonine kinase 1 [25]. Moreover, miR-215-5p mediated suppression of mesothelioma progression via the MDM2-p53-signaling axis [26] and hepatocellular carcinoma progression suppression via miR–215–PCAT-1 axis were reported recently [27]. On the other hand, miR-215-5p was reported to act as an oncogene in several studies. A study from 2016 found the up-regulation of this miRNA to be closely associated with high-grade glioma and poor overall survival, enhanced migration, and invasion of glioma cells via targeting retinoblastoma tumor suppressor gene 1 (RB1) [28]. Moreover, targeting of RB1 by this miRNA was also reported in gastric cancer [29]. Another study proposed miR-215-5p function as an oncogene in gastric cancer by targeting RUNX1 tumor suppressor [30]. Moreover, several studies found miR-215-5p to be a promising diagnostic and prognostic biomarker in CRC, ovarian cancer, or HCV-associated hepatocellular carcinoma [31,32,33]. Thus, it seems that miR-215-5p may act as both tumor suppressor and an oncogene based on the microenvironment and different mechanisms of action. Nevertheless, it is usually reported to be an important tumor suppressor in CRC, and its low levels are associated with worse prognosis and disease progression. Despite the growing number of studies analyzing the role of this miRNA in CRC pathogenesis and metastases formation, precise mechanisms are still not understood.

During our previous research, we have observed significantly down-regulated levels of miR-215-5p not only in primary tumors of CRC patients but also in corresponding liver metastases. Thus, we decided to use the stable transfection of miR-215-5p into three different CRC cell lines to study the effects of its supplementation on the metastatic process. The results proved that higher levels of miR-215-5p significantly reduced the clonogenic potential of HCT-116 and RKO cells. A similar effect on clonogenicity was previously observed in HCT-116 cells and associated with loss of stemness [10]. Contrary, the opposite effects were observed in the case of HCT-15 cells, where increased levels of miR-215-5p led to enhanced clonogenic potential, which implies lower anchorage dependency of these cells and might be necessary to perform anchorage-independent colony forming assay to assess the clonogenic potential of this cell line more accurately. However, supplementation with miR-215-5p led to a significantly smaller diameter of the colonies in the case of all three cell lines.

Additionally, we performed cell cycle, apoptosis and cell death analyses. Cell cycle arrest in G1/G0 phase was observed in RKO cells transfected with miR-215-5p, while HCT-15 and HCT-116 cells overexpressing this miRNA showed significant cell cycle arrest in S phase compared to control cells. Analysis of early apoptosis showed significant results only in the case of HCT-15 cell line. However, the number of death cells increased significantly in HCT-116 and RKO cell lines overexpressing miR-215-5p. These results are in accordance with our previous data [20], however, there are some inconsistencies that might be caused by a different type of transfection and dramatically different expression levels of miR-215-5p reached by each type of transfection. Another critical factor might be the artificiality of in vitro assays. Our in vivo and NGS results indicate that miR-215-5p influences cells on the level of the cell to cell interaction, and more microenvironment-oriented studies might be necessary to uncover the miR-215-5p manner of action fully.

Subsequently, we observed that higher levels of miR-215-5p lead to significant inhibition of cell migration and invasiveness in HCT-15 cells. Surprisingly, HCT-116 cells showed increased migratory capacity but evinced a decreasing trend of invasiveness after miR-215-5p overexpression; however, the changes were not statistically significant. MiR-215-5p was previously described to regulate genes involved in cell to cell adhesion and ECM interactions; thus, we hypothesize that changes in expression of these genes might bias results of transwell migration/invasion assay. Previously, we confirmed the ectopic overexpression of miR-215-5p induced by transient transfection to inhibit the migration of HCT-116 cells [20]. Factor influencing the transwell migration/invasion assay of HCT-116 might be a diverse amount of cells used for the experiments. MiR-192, a miRNA from the same family with high homology to miR-215-5p, was reported to inhibit proliferation, migration, and invasion of cell line HCT-116. Recently, similar results were observed by Xu et al. [34] using the HT-29 cell line when enhanced levels of miR-215-5p suppressed cell migration and invasion via direct targeting of stearoyl-CoA desaturase, a key enzyme involved in β-catenin signaling pathway [35]. In 2008, Braun et al. [9] found that miR-215-5p has an effect on cellular adhesion and induced cell detachment. In addition, two different lncRNAs – UICLM and FTX—were identified as important miR-215-5p sponges regulating ZEB2 expression and vimentin phosphorylation, thus contributing to CRC progression [14,16].

In vivo tumorigenicity assay confirmed tumor suppressor effects of miR-215-5p in CRC in all three cell lines as tumors of cells with increased miR-215-5p had smaller weight and volume. We observed statistically significant differences in tumor weight in HCT-116 and HCT-15 cell lines. Statistically significant differences in tumor volume were observed in RKO and HCT-15 cell lines. These results comply with literature and inhibition of tumor growth by miR-215-5p overexpression in vivo was observed in ovarian cancer [36], hepatocellular cancer [27], and non-small cell lung carcinoma [22]. The intra-splenic metastatic model further confirmed tumor suppressor properties of miR-215-5p as overexpression of this miRNA resulted in a significantly decreased number of liver metastases formed by HCT-116, RKO, and HCT-15 cells.

Overall, observed effects of supplementation with miR-215-5p in CRC cells in vitro and especially in vivo support the tumor suppressor role of this miRNA in CRC and indicate that future modulation of miR-215-5p activity could be a promising strategy in CRC treatment. However, the mechanism of its involvement in metastases formation and disease progression remains unclear. We decided to perform the transcriptome profiling of cells with upregulated levels of miR-215-5p with the aim to identify potential direct or indirect targets of this miRNA and further describe its involvement in various cellular processes and molecular pathways.

Since the tumorigenic and metastatic potential of HCT-15 showed the strongest dependence on miR-215-5p expression, HCT-15-miR-215-5p and HCT-15-control cells were used for transcriptome profiling. Differential expression analysis identified 8,847 mRNAs with significantly deregulated expression, and subsequent KEGG pathway analysis revealed 22 different signaling pathways connected to miR-215-5p deregulation. In total, 351 mRNAs significantly influenced by miR-215-5p transfection were associated with metastatic pathways and processes or adhesion molecules, including focal adhesion, extracellular matrix-receptor interactions, adherent junctions, tight junctions, Wnt signaling pathway, angiogenesis, the pluripotency of stem cells, proteoglycans, and other adhesion molecules. From those, seven mRNAs (MSN, CREB5, CTNNBIP1, PRKACB, MYLK, ACVR2A, CBL) were predicted to be potential direct targets of miR-215-5p based on the TargetScan 7.2 database, while the others are probably the indirect targets of this miRNA. We performed RT-qPCR validation and found out all seven genes to be significantly deregulated in subcutaneous tumors generated with HCT-15 cells stably transfected with miR-215-5p compared to control cells tumors. While the expression of CTNNBIP1, ACVR2A, MYLK, and PRKACB was significantly lower in these tumors in comparison to tumors generated with control cells, significantly higher expression levels of MSN, CREB5, and CBL were detected. These data indicate that CTNNBIP1, ACVR2A, MYLK, and PRKACB could be potential direct targets of miR-215-5p. Moreover, CTNNBIP1 was significantly upregulated in primary tumor tissue and metastatic liver tissue of CRC patients compared to adjacent colon tissue. Interestingly, CTNNBIP1 has been previously confirmed to be a direct target of miR-215-5p in various diagnoses, including human glioma [37], diabetic nephropathy, or atherosclerosis [38], and promoting the tumor growth through Wnt/β-catenin signaling pathway. In nephroblastomas, ACVR2B was found to be a direct target of miR-215-5p. Activins are involved in the tumor growth factor β (TGF-β) signaling pathway and act on cell growth and differentiation. Importantly, undifferentiated mesenchyme was proved to be negative for ACVR2B expression, which is in agreement with higher levels of miR-215 in undifferentiated tissues [39]. MYLK upregulated expression was reported to correlate with the severity of inflammatory bowel disease [40], a major risk for the development of gastrointestinal malignancies [41]. Moreover, inhibition of MYLK was reported to induce apoptosis and reduce tumor growth in vivo [42]. PRKCB overexpression was found to attenuate autophagy, an important pathway in diseases such as neurodegeneration and cancer [43]. MSN and CREB5 were reported to have upregulated expression in CRC tissue and promote invasiveness [44,45]. Lastly, c-Cbl was shown to modulate Wnt signaling pathway and higher expression in tumors correlated with longer overall survival of patients with mCRC [46]. Recently, expression of leucine-rich repeat-containing G-protein coupled receptor 5 (LGR5) has been clearly associated with the initiation of primary intestinal cancers as well as CRC-derived liver metastases [47]. LGR5 is an essential member of the Wnt signaling pathway and a well-accepted marker of non-neoplastic intestinal stem cells [48], and its downregulated levels were observed in our study in HCT-15 cells transfected with miR-215-5p. Ullmann et al. [49] observed a significant downregulation of this protein under hypoxic conditions, while the expression of miR-215-5p was upregulated under hypoxia. In addition, overexpression of miR-215-5p decreased colon tumor-initiating cells activity. As there are no reported binding sites for this miRNA at 3′UTR of LGR, it is highly probable that miR-215-5p represses a transcriptional coactivator of LGR5. 

Altogether, our findings are in high compliance with previous studies and support the relevance of miR-215-5p to CRC pathogenesis. The involvement of this miRNA in CRC metastasis is highly probable through numerous signaling pathways, especially those associated with focal adhesion, extracellular matrix-receptor interactions, Wnt signaling pathway, or pluripotency of stem cells. Nevertheless, the particular molecules and direct targets of miR-215-5p involved in this process and responsible for disease progression must be elucidated in the curse of subsequent studies.

## 4. Materials and Methods

### 4.1. Cell Lines and Cell Culture

Three stable human CRC cell lines HCT-116 (ATCC^®^ CCL-247™), RKO (ATCC^®^ CRL-2577™), and HCT-15 (ATCC^®^ CCL-225™) purchased from ATCC (Manassas, VI, USA) were cultivated according to ATCC recommendations. HCT-116 cells were cultured in McCoy’s 5A medium (Sigma Aldrich, St. Louis, MI, USA) supplemented with 10% fetal bovine serum, 2 mM GlutaMAX™ Supplement, and 100 µg/mL^−1^ penicillin, 100 µg/Ml^−1^ streptomycin in 5% CO_2_ at 37 °C. RKO cells were cultured in Minimum Essential Medium supplemented with 10% fetal bovine serum, and 100 µg/mL^−1^ penicillin, 100 µg/mL^−1^ streptomycin, 0.1 mM non-essential amino acids, 2 mM GlutaMAX™ Supplement, 1 mM sodium pyruvate (Invitrogen, Gibco, Carlsbad, CA, USA) in 5% CO_2_ at 37 °C. HCT-15 cells were cultivated in RPMI-1640 medium supplemented with 10% fetal bovine serum, 2 mM GlutaMAX™ Supplement, and 100 µg/mL^−1^ penicillin, 100 µg/mL^−1^ streptomycin in 5% CO_2_ at 37 °C. All media and supplements used were obtained from Gibco (Gibco, Thermo Fisher Scientific, Waltham, MA, USA). All cell lines were regularly tested with MycoAlert (Lonza Group Ltd., Basel, Switzerland) to ensure the absence of mycoplasma contamination. Authentication of cell lines was done by comparing STR (short tandem repeat) sequences obtained from actual cell lines as determined by Generi Biotech (Hradec Kralove, Czech Republic) with data publicly available (ATCC, ECACC—European Collection of Authenticated Cell Cultures). Recent STR analysis has been performed within six months before the beginning or in the course of the experiments for all cell lines.

### 4.2. Patient Samples

For the purpose of validation of mRNAs potentially regulated by miR-215-5p and identified by transcriptome analysis, CRC patient samples were included in the study. We used fresh frozen tissue samples of primary CRC tissue, paired CRC liver metastatic tissue, and adjacent mucosa collected from ten patients. All patients were treated at Masaryk Memorial Cancer Institute in Brno and signed an informed consent form and study was approved by the local Ethics Board at Masaryk Memorial Cancer Institute.

### 4.3. Stable Transfection of miR-215

For functional studies, we stably transfected cell lines HCT-116, RKO, and HCT-15 using TurboFectin 8.0 and pCMV-miR-215 vector (OriGene Technologies, Rockville, MD, USA, CAT#: SC400278) and empty pCMV-MIR vector (CAT#: PCMVMIR). The first selection was performed using 400 µg/mL^−1^ of G418 antibiotics (Sigma Aldrich, Saint Louis, MO, USA). The second selection and isolation of transfected clones were based on green fluorescent protein fluorescence and using BD FACS Canto II Cell Analyzer (BD Biosciences, New Jersey, NJ, USA). Clones for further studies were selected according to the expression of miR-215-5p, HOXB9, and epiregulin (EREG) measured by RT-qPCR.

### 4.4. Colony Forming Aassay

The colony-forming assay was performed using six-well plates and tumor cells stably transfected with miR-215-5p or control vector were seeded in a concentration of 250 cells per well. After 12–14 days, colonies were stained with crystal violet blue solution (Sigma-Aldrich). Plates were scanned, and colonies counted and analyzed by GelCount (Oxford Optronix, Abingdon, UK).

### 4.5. Cell Cycle, Apoptosis and Cell Death analysis

For cell cycle analysis, cells were trypsinized and fixed in ice cold 70% ethanol for 30 min and then stained with propidium iodide (PI) solution (1 μg/μL PI and 0.1 mg/mL RNaseA (Sigma Aldrich, St. Louis, MO, USA)) and incubated on ice for 20 min. Apoptosis and cell death analysis was performed using Annexin V, Alexa Fluor™ 647 conjugate and Annexin Binding Buffer (5X), for flow cytometry (Invitrogen, Gibco, Carlsbad, CA, USA) according to manufacturer’s instructions. Cells were analyzed using BD FACS Verse flowcytometer (BD Biosciences, San Jose, MO, USA) and the percentage of cells in each phase of the cell cycle and viability and apoptosis were determined using BD FACSuite software (BD Biosciences, San Jose, MO, USA).

### 4.6. Transwell Migration and Invasiveness Assay

Transwell invasiveness assay was performed using Corning^®^ BioCoat™ Matrigel^®^ Invasion Chambers with 8.0 µm PET Membrane (Corning Incorporated, Corning, NY, USA). Transwell migration assay and control for invasiveness assay were done using Corning^®^ BioCoat™ Control Inserts with 8.0 µm PET Membrane (Corning Incorporated, Corning, NY, USA). Cells were previously starved in media supplemented with 2% FBS for 48 h and then seeded in concentrations HCT-116 (5 × 10^4^ cells/well), RKO (7.5 × 10^4^ cells/well), and HCT-15 (5 × 10^4^ cells/well) and incubated for 24 h in 5% CO_2_ at 37 °C. Staining was performed with Hoechst 33,342 (Invitrogen), and migrated/invaded cells were visualized using a confocal microscope ZEISS LSM 700 and ZEISS ZEN Software (Carl Zeiss AG, Oberkochen, Germany). Subsequently, cells were counted using ImageJ software (Wayne Rasband, NIH, Bethesda, MD, USA).

### 4.7. Animal Models

In vivo functional studies were performed in NOD SCID gamma (NSG) mice (obtained from The Jackson Laboratory, Bar Harbor, ME, USA) housed and monitored in individually ventilated cage system (Techniplast, Buguggiate, Italy) with ad libitum access to water and feeding. Animal experiments were performed in accordance with national and EU animal welfare legislation, and all procedures were approved by institutional (Masaryk University, Brno, Czech Republic) and national ethics committees. Identification number of project MSMT-9643/2017-3.

### 4.8. Tumorigenicity Assay

Eighteen NSG mice (males, 12–16 weeks old, weighing 25‒31 g, 6 for each cell line) were used for subcutaneous injections of selected stable transfected clones. For procedures involving subcutaneous injection, mice were anesthetized by intraperitoneal injection of xylazine (100 mg/kg of weight)/ketamine (10 mg/kg of weight). The injected volume of cell suspension was 2.5 × 10^6^ cells per 100 µL of PBS. During the experiment, tumor growth and animal behavior were individually monitored. Mice were sacrificed, and an autopsy was performed according to palpation tests—after 29 days (HCT-116), after 22 days (RKO), and after 15 days (HCT-15).

### 4.9. Intra-Splenic Metastatic Model

For procedures involving surgery, 27 mice were anesthetized (HCT-116 8 animals, RKO 11 animals, HCT-15 8 animals) by intraperitoneal injection of xylazine (100 mg/kg of weight)/ketamine (10 mg/kg of weight). Suspension of 5 × 10^4^ cells in 3µL of PBS was injected at the inferior pole of the spleen using Hamilton syringe and a 26 s needle. Whitening of the spleen and blood vessels was observed upon injection. Five minutes after injection, mice underwent splenectomy through ligature of the pancreas and splenic vessels and complete resection of the spleen. After surgical intervention, Ketamine (2 mg/kg s.c.) analgesia was administered into the skin fold. Animals were sacrificed, and an autopsy performed after 25 days (HCT-116), after 23 days (RKO), and after 25 days (HCT-15). Removed livers were fixed in 10% formalin and used for the preparation of formalin-fixed paraffin-embedded samples. Liver metastases were analyzed semi-quantitatively.

### 4.10. RNA Isolation

Total RNA enriched with a fraction of small RNAs was isolated using mirVana miRNA Isolation Kit (Ambion, Austin, TX, USA) in the case of subcutaneous tumors from NSG mice and fresh frozen tissue samples from patients and with Direct-zol RNA Microprep Kit (Zymo Research, Irvine, CA, USA) in the case of cell lines according to the manufacturer’s instructions. Quality and quantity of isolated RNA were measured spectrophotometrically using NanoDrop 2000c (Thermo Fisher Scientific, Waltham, MA, USA) and fluorometrically using Qubit 2.0 and Qubit BR RNA Assay Kit (Thermo Fisher Scientific). RNA integrity numbers of isolated RNAs used for the preparation of cDNA libraries were measured using the TapeStation Instrument 2000 and RNA Screen Tape (Agilent Technologies, Santa Clara, CA, USA).

### 4.11. Reverse Transcription and Quantitative Real-Time PCR

MiRNA reverse transcription was performed using 6,67 ng of total RNA, gene-specific primers (hsa-miR-215-5p; ID 000518, RNU48; ID 001006), and TaqMan™ MicroRNA Reverse Transcription Kit according to the TaqMan MicroRNA Assay protocol (Applied Biosystems, Foster City, CA, USA). MiRNA RT-qPCR was performed using TaqMan™ Universal Master Mix II, no UNG (Applied Biosystems) according to manufacturer’s recommendations. Gene expression reverse transcription was performed using 1000 ng of total RNA and Capacity cDNA Reverse Transcription Kit (Applied Biosystems, Foster City, CA, USA) according to manufacturer’s protocol. Gene expression RT-qPCR master mix was prepared using TaqMan™ Gene Expression Master Mix (Applied Biosystems, Foster City, CA, USA) and gene specific primers TaqMan™ Gene Expression Assays (MYLK; ID Hs00364926_m1, ACVR2A; ID Hs00155658_m1, CBL; ID Hs01011446_m1, MSN; ID Hs00741306_mH, PMM1; ID Hs00160195_m1, GAPDH; ID Hs02758991_g1, PRKACB; ID Hs01086757_m1, CTNNBIP1; ID Hs00172016_m1, and CREB5; ID Hs00191719_m1), (Applied Biosystems, Foster City, CA, USA). RT-qPCR was performed using the QuantStudio 12K Flex Real-Time PCR System (Applied Biosystems).

### 4.12. Transcriptome Profiling

For the purpose of evaluation of alterations on transcriptome level induced by increased expression of miR-215-5p, RNA-seq was performed. Three samples of RNA isolated from HCT-15 clone stably transfected with pCMV-miR-215 and three samples of RNA isolated from HCT-15 clone stably transfected with pCMV-MIR vector were used for cDNA library preparation. RNA integrity numbers of samples were 9.4–10, and the input of RNA into polyA selection was 1000 ng/50 µL. PolyA selection was performed using NEBNext^®^ Poly(A) mRNA Magnetic Isolation Module (New England Biolabs, Beverly, MA, USA). Preparation of cDNA libraries was done using NEBNext^®^ Ultra™ II Directional RNA Library Prep Kit for Illumina^®^ (New England Biolabs, USA) and NEBNext^®^ Multiplex Oligos for Illumina^®^ (New England Biolabs). The quantity of cDNA libraries was measured using Qubit and Qubit hs DNA Assay Kit (Thermo Fisher Scientific), and the length of cDNA libraries was measured using TapeStation Instrument 2000 and High Sensitivity D1000 ScreenTape (Agilent Technologies). Concentrations of cDNA libraries were ranging between 20 and 228 nM and length between 303 and 375 bp. Libraries were diluted to 4 nM and pooled into the final 4 nM pool of cDNA libraries used for sequencing. Sequencing was done using the NextSeq 500/550 High Output Kit v2 and NextSeq 500/550 instrument (Illumina, San Diego, CA, USA) and the sequencing setup was single read, 80 cycles.

### 4.13. Statistical Analysis

Expression levels of miR-215-5p measured by RT-qPCR were normalized using RNU48 as endogenous control and formula Ct(miR-215-5p normalized expression)= 2^-(ΔCt miR-215-5p duplicate—ΔCt RNU48 duplicate).^ Expression levels of MYLK, ACVR2A, MSN, CBL, PRKCB, CREB5, and CTNNBIP1 measured by RT-qPCR were normalized using GAPDH in subcutaneous tumors generated in mice and PMM1 in patient samples using formula Ct(Gene × normalized expression) = 2^-(ΔCt Gene x duplicate—ΔCt PMM1/GAPDH duplicate)^. Unpaired parametric two-sided t-test was used to analyze colony formation assay, transwell migration/invasive assay, expression of miR-215, weight and volume of subcutaneous tumors, and the number of liver metastases, and expression data from subcutaneous tumors. All in vitro analyses were performed at least three times. Expression data from paired patient samples were analyzed using Wilcoxon matched-pairs signed rank test. Analyses were done using GraphPad Prism 8 (GraphPad Software, San Diego, CA, USA). P-values of less than 0.05 were considered statistically significant.

### 4.14. Processing of RNA-Seq Data

The raw sequencing images from Illumina NextSeq 550 were demultiplexed and converted to fastq format using bcl2fastq (version 2.20.0). Generated reads were single-end and 100 nucleotides long. FastQC (version 0.11.9) [50] used to examine the quality of raw reads. Adapter sequences in raw sequencing data were trimmed using Trimmomatic (version 0.39) [51] and reads shorter than 35 nt were removed. The alignment of pre-processed reads against the human genome was done with splicing aware tool STAR (version 2.7.0d) [52] using Ensembl genome assembly GRCh38 and gene annotation GRCh38.91. Qualimap (version 2.2.2a) [53] and FastQ Screen (version 0.13.0) [54] were used to check mapping quality. Reads per gene were counted with RSEM (version 1.3.1) [55]. Differential gene expression analysis was carried out with R/Bioconductor package DESeq2 (version 1.24.0) [56]. The R/Bioconductor package clusterProfiler [57] was used to identify enriched KEGG pathways [58] and the most over-represented Gene Ontology terms [59,60] based on a list of top DEG genes with DESeq2 adjusted *p*-value < 0.05 and fold change > 2.

## 5. Conclusions

MiR-215-5p has a vital role in CRC pathogenesis, including the metastatic process. We hypothesize this miRNA to regulate the metastatic potential of CRC cells via modulation of divergent molecular pathways including extracellular-matrix-receptor interaction, focal adhesion, Wnt signaling pathway, and pluripotency of stem cells. However, specific targets of miR-215-5p that contribute to the formation of distant metastases and thus the progression of the disease must be further elucidated.

## Figures and Tables

**Table 1:** Top 10 KEGG enriched pathways associated with miR-215-5p overexpression in HCT-15 cell line.

KEGG ID	Number of Genes	Adjusted *p*-Value	Description
hsa04512	45	1.9955 × 10^−5^	ECM-receptor interaction
hsa04510	90	2.7430 × 10^−5^	Focal adhesion
hsa05412	38	1.9617 × 10^−3^	Arrhythmogenic right ventricular cardiomyopathy
hsa04151	127	2.0126 × 10^−3^	PI3K-Akt signaling pathway
hsa00980	31	2.0813 × 10^−3^	Metabolism of xenobiotics by cytochrome P450
hsa04950	14	3.3039 × 10^−3^	Maturity onset diabetes of the young
hsa05200	186	3.9291 × 10^−3^	Pathways in cancer
hsa05217	32	4.6798 × 10^−3^	Basal cell carcinoma
hsa00982	28	4.7884 × 10^−3^	Drug metabolism - cytochrome P450
hsa04060	91	4.7884 × 10^−3^	Cytokine-cytokine receptor interaction

**Table 2:** Top 10 GO terms associated with miR-215-5p overexpression in HCT-15 cell line.

**Top 10 GO Molecular Functions Terms**				
**GO ID**	**Number of Genes**	**Background Genes**	**Adjusted *p*-Value**	**Term**
GO:0005509	235	544	3.5724 × 10^−7^	Calcium ion binding
GO:0019838	58	110	1.0470 × 10^−3^	Growth factor binding
GO:0003779	143	336	1.0470 × 10^−3^	Actin binding
GO:0005201	65	129	1.0470 × 10^−3^	Extracellular matrix structural constituent
GO:0098772	464	1282	1.2080 × 10^−3^	Molecular function regulator
GO:0015267	122	282	1.2080 × 10^−3^	Channel activity
GO:0022803	122	282	1.2080 × 10^−3^	Passive transmembrane transporter activity
GO:0004714	28	44	1.6820 × 10^−3^	Transmembrane receptor protein tyrosine kinase activity
GO:0005102	417	1150	2.3635 × 10^−3^	Signaling receptor binding
GO:0019199	34	59	3.0812 × 10^−3^	Transmembrane receptor protein kinase activity
**Top 10 GO biological processes terms**				
**GO ID**	**Number of Genes**	**Background Genes**	**Adjusted *p*-Value**	**Term**
GO:0048856	1729	4694	3.4494 × 10^−25^	Anatomical structure development
GO:0032501	2074	5804	2.8832 × 10^−24^	Multicellular organismal process
GO:0048731	1451	3876	3.4175 × 10^−23^	System development
GO:0007275	1593	4320	6.2510 × 10^−23^	Multicellular organism development
GO:0032502	1811	5012	2.1700 × 10^−22^	Developmental process
GO:0030154	1225	3234	1.0946 × 10^−20^	Cell differentiation
GO:0009653	858	2152	3.8805 × 10^−20^	Anatomical structure morphogenesis
GO:0040011	607	1436	6.4324 × 10^−20^	Locomotion
GO:0051239	1005	2592	9.4443 × 10^−20^	Regulation of multicellular organismal process
GO:0048869	1266	3380	1.3098 × 10^−19^	Cellular developmental process
**Top 10 GO cellular components terms**				
**GO ID**	**Number of Genes**	**Background Genes**	**Adjusted *p*-Value**	**Term**
GO:0071944	1512	3934	2.7470 × 10^−32^	Cell periphery
GO:0005886	1484	3853	2.7470 × 10^−32^	Plasma membrane
GO:0044459	773	1916	4.0527 × 10^−20^	Plasma membrane part
GO:0044425	1723	4911	8.8179 × 10^−15^	Membrane part
GO:0031224	1351	3755	1.7922 × 10^−14^	Intrinsic component of membrane
GO:0098590	412	976	2.1436 × 10^−13^	Plasma membrane region
GO:0016021	1300	3640	1.2325 × 10^−12^	Integral component of membrane
GO:0005576	1188	3298	1.7219 × 10^−12^	Extracellular region
GO:0016020	2454	7347	5.7937 × 10^−12^	Membrane
GO:0005911	177	373	4.0630 × 10^−10^	Cell-cell junction

## Supplementary Materials

The following are available online at https://www.mdpi.com/2072-6694/12/12/3518/s1, Supplementary File 1 (.png) Figure with graph with miR-215-5p expression in selected clones of HCT-116, RKO, and HCT-15 cells. Supplementary File 2 (.png) Cell cycle analysis, apoptosis and cell death analysis of CRC cell lines stably transfected with miR-215-5p and control cell lines. Supplementary File 3 (.xlsx): A list of 8,847 mRNAs with significantly (*p* adjust. < 0.001) deregulated expression in miR-215-5p transfected HCT-15 cells compared to HCT-15 control cells. Supplementary File 4 (.xlsx): A list of 273 mRNAs significantly influenced by miR-215-5p overexpression (*p* < 0.05; at least 100 raw reads in all 3 samples from one condition—miR-215-5p or mock—in average) and associated with metastatic pathways and processes, adhesion molecules, angiogenesis, or pluripotency of stem cells. Supplementary File 5 (.docx): A list of mRNAs significantly deregulated in HCT-15 cells overexpressing miR-215-5p and associated with focal adhesion based on the KEGG pathway analysis. Supplementary File 6 (.docx): A list of mRNAs significantly deregulated in HCT-15 cells overexpressing miR-215-5p and associated with ECM-receptor interaction based on the KEGG pathway analysis.

## List of Abbreviations

microRNAs: miRNAs, CRC: colorectal cancer, CDX1: caudal type homeobox 1, NF-κB: nuclear factor κB, NGS: next generation sequencing, NSG: NOD SCID gamma, EREG: epiregulin, ECM: extra-cellular matrix, ZEB2: zinc finger E-box binding homeobox 2, EMT: epithelial-mesenchymal transition, MMP16: matrix metalloproteinase-16, RB1: retinoblastoma tumor suppressor gene 1, CTNNBIP1: catenin beta interacting protein 1, ACVR2B: activin A receptor type 2B, MSN: Moesin; CREB5: cAMP responsive element binding protein 5; PRKACB: Protein kinase cAMP-activated catalytic subunit beta; MYLK: Myosin Light Chain Kinase; ACVR2A: A receptor type 2B; CBL: Cbl proto-oncogene; TGF-β: tumor growth factor β, LGR5: leucine-rich repeat-containing G-protein coupled receptor 5, LFC: logarithmic fold change, SE: standard error.

## References

[1] Bray F., Ferlay J., Soerjomataram I., Siegel R.L., Torre L.A., Jemal A. (2018). Global cancer statistics 2018: GLOBOCAN estimates of incidence and mortality worldwide for 36 cancers in 185 countries. CA Cancer J. Clin..

[2] A Issa I., Noureddine M. (2017). Colorectal cancer screening: An updated review of the available options. World J. Gastroenterol..

[3] Engstrand J., Nilsson H., Strömberg C., Jonas E., Freedman J. (2018). Colorectal cancer liver metastases—A population-based study on incidence, management and survival. BMC Cancer.

[4] Balchen V., Simon K. (2016). Colorectal cancer development and advances in screening. Clin. Interv. Aging.

[5] Molinari C., Marisi G., Passardi A., Matteucci L., De Maio G., Ulivi P. (2018). Heterogeneity in Colorectal Cancer: A Challenge for Personalized Medicine?. Int. J. Mol. Sci..

[6] Lai E.C. (2002). Micro RNAs are complementary to 3′ UTR sequence motifs that mediate negative post-transcriptional regulation. Nat. Genet..

[7] Ding L., Lan Z., Xiong X., Ao H., Feng Y., Gu H., Yu M., Cui Q. (2018). The Dual Role of MicroRNAs in Colorectal Cancer Progression. Int. J. Mol. Sci..

[8] Khella H., Bakhet M., Allo G., Jewett M., Girgis A., Latif A., Von Both I., Bjarnason G., Yousef G.M. (2013). miR-192, miR-194 and miR-215: A convergent microRNA network suppressing tumor progression in renal cell carcinoma. Carcinogenesis.

[9] Braun C.J., Zhang X., Savelyeva I., Wolff S., Moll U.M., Schepeler T., Ørntoft T.F., Andersen C.L., Dobbelstein M. (2008). p53—Responsive MicroRNAs 192 and 215 Are Capable of Inducing Cell Cycle Arrest. Cancer Res..

[10] Jones M.F., Hara T., Francis P., Li X.L., Bilke S., Zhu Y., Pineda M., Subramanian M., Bodmer W., Lal A. (2015). The CDX1-microRNA-215 axis regulates colorectal cancer stem cell differentiation. Proc. Natl. Acad. Sci. USA.

[11] Lu H., Lei X., Liu J., Klaassen C. (2017). Regulation of hepatic microRNA expression by hepatocyte nuclear factor 4 alpha. World J. Hepatol..

[12] Mechtler P., Singhal R., Kichina J.V., Bard J.E., Buck M.J., Kandel E.S. (2015). MicroRNA analysis suggests an additional level of feedback regulation in the NF-κB signaling cascade. Oncotarget.

[13] Hu J., Sun T., Wang H., Chen Z., Wang S., Yuan L., Liu T., Li H.-R., Wang P., Feng Y. (2016). MiR-215 Is Induced Post-transcriptionally via HIF-Drosha Complex and Mediates Glioma-Initiating Cell Adaptation to Hypoxia by Targeting KDM1B. Cancer Cell.

[14] Chen D.-L., Lu Y.-X., Zhang J.-X., Wei X.-L., Wang F.-H., Zeng Z.-L., Pan Z.-Z., Yuan Y.-F., Pelicano H., Chiao P.J. (2017). Long non-coding RNA UICLM promotes colorectal cancer liver metastasis by acting as a ceRNA for microRNA-215 to regulate ZEB2 expression. Theranostics.

[15] Kong X., Duan Y., Sang Y., Li Y., Zhang H., Liang Y., Liu Y., Zhang N., Yang Q. (2019). LncRNA-CDC6 promotes breast cancer progression and function as ceRNA to target CDC6 by sponging microRNA-215. J. Cell. Physiol..

[16] Yang Y., Zhang J., Chen X., Xu X., Cao G., Li H., Wu T. (2018). LncRNA FTX sponges miR-215 and inhibits phosphorylation of vimentin for promoting colorectal cancer progression. Gene Ther..

[17] Vychytilova-Faltejskova P., Slaby O. (2019). MicroRNA-215: From biology to theranostic applications. Mol. Asp. Med..

[18] Necela B.M., Carr J.M., Asmann Y.W., Thompson E.A. (2011). Differential Expression of MicroRNAs in Tumors from Chronically Inflamed or Genetic (APCMin/+) Models of Colon Cancer. PLoS ONE.

[19] Faltejskova P., Svoboda M., Srutova K., Mlcochova J., Besse A., Nekvindova J., Radova L., Fabian P., Slaba K., Kiss I. (2012). Identification and functional screening of microRNAs highly deregulated in colorectal cancer. J. Cell. Mol. Med..

[20] Vychytilova-Faltejskova P., Merhautova J., Machackova T., Gutierrez-Garcia I., Garcia-Solano J., Radova L., Brchnelova D., Slaba K., Svoboda M., Halamkova J. (2017). MiR-215-5p is a tumor suppressor in colorectal cancer targeting EGFR ligand epiregulin and its transcriptional inducer HOXB9. Oncogenesis.

[21] Li S., Gao J., Gu J., Yuan J., Hua D., Shen L. (2013). MicroRNA-215 inhibits relapse of colorectal cancer patients following radical surgery. Med. Oncol..

[22] Hou Y., Zhen J., Xu X., Zhen K., Zhu B., Pan R., Zhao C. (2015). miR-215 functions as a tumor suppressor and directly targets ZEB2 in human non-small cell lung cancer. Oncol. Lett..

[23] Yao Y., Shen H., Zhou Y., Yang Z., Hu T. (2018). MicroRNA-215 suppresses the proliferation, migration and invasion of non-small cell lung carcinoma cells through the downregulation of matrix metalloproteinase-16 expression. Exp. Ther. Med..

[24] Gao J.B., Zhu M.N., Zhu X.L. (2019). miRNA-215-5p suppresses the aggressiveness of breast cancer cells by targeting Sox9. FEBS Open Bio..

[25] Yao J., Zhang P., Li J., Xu W. (2017). MicroRNA-215 acts as a tumor suppressor in breast cancer by targeting AKT serine/threonine kinase 1. Oncol. Lett..

[26] Singh A., Bhattacharyya N., Srivastava A., Pruett N., Ripley R.T., Schrump D.S., Hoang C.D. (2019). MicroRNA-215-5p Treatment Suppresses Mesothelioma Progression via the MDM2-p53-Signaling Axis. Mol. Ther..

[27] Ren Y., Shang J., Li J., Liu W., Zhang Z., Yuan J., Yang M. (2017). The long noncoding RNA PCAT-1 links the microRNA miR-215 to oncogene CRKL-mediated signaling in hepatocellular carcinoma. J. Biol. Chem..

[28] Wei Y., Sun J., Li X. (2016). MicroRNA-215 enhances invasion and migration by targeting retinoblastoma tumor suppressor gene 1 in high-grade glioma. Biotechnol. Lett..

[29] Chen Z., Liu K., Li L., Chen Y., Du S. (2017). miR-215 promotes cell migration and invasion of gastric cancer by targeting Retinoblastoma tumor suppressor gene 1. Pathol. Res. Pract..

[30] Li N., Zhang Q.-Y., Zou J.-L., Li Z.-W., Tian T.-T., Dong B., Liu X.-J., Ge S., Zhu Y., Gao J. (2016). miR-215 promotes malignant progression of gastric cancer by targeting RUNX1. Oncotarget.

[31] Mamdouh S., Khorshed F.E., Aboushousha T., Hamdy H., Diab A., Seleem M., Saber M. (2017). Evaluation of Mir-224, Mir-215 and Mir-143 as Serum Biomarkers for HCV Associated Hepatocellular Carcinoma. Asian Pac. J. Cancer Prev..

[32] Karaayvaz M., Pal T., Song B., Zhang C., Georgakopoulos P., Mehmood S., Burke S., Shroyer K., Ju J. (2011). Prognostic Significance of miR-215 in Colon Cancer. Clin. Color. Cancer.

[33] Agostini A., Brunetti M., Davidson B., Tropé C.G., Eriksson A.G.Z., Heim S., Panagopoulos I., Micci F. (2018). The microRNA miR-192/215 family is upregulated in mucinous ovarian carcinomas. Sci. Rep..

[34] Xu X., Ding Y., Yao J., Wei Z., Jin H., Chen C., Feng J., Ying R. (2020). miR-215 Inhibits Colorectal Cancer Cell Migration and Invasion via Targeting Stearoyl-CoA Desaturase. Comput. Math. Methods Med..

[35] Mauvoisin D., Charfi C., Lounis M.A., Rassart E., Mounier C. (2012). Decreasing stearoyl-CoA desaturase-1 expression inhibits βcatenin signaling in breast cancer cells. Cancer Sci..

[36] Lin Y., Jin Y., Xu T., Zhou S., Cui M. (2017). MicroRNA-215 targets NOB1 and inhibits growth and invasion of epithelial ovarian cancer. Am. J. Transl. Res..

[37] Tong Y.-Q., Liu B., Zheng H.-Y., Gu J., Liu H., Li F., Tan B.-H., Hartman M., Song C., Li Y. (2015). MiR-215, an activator of the CTNNBIP1/β-catenin pathway, is a marker of poor prognosis in human glioma. Oncotarget.

[38] Mu J., Pang Q., Guo Y.-H., Chen J.-G., Zeng W., Huang Y.-J., Zhang J., Feng B. (2013). Functional Implications of MicroRNA-215 in TGF-β1-Induced Phenotypic Transition of Mesangial Cells by Targeting CTNNBIP1. PLoS ONE.

[39] Senanayake U., Das S., Vesely P., Alzoughbi W., Fröhlich L.F., Chowdhury P., Leuschner I., Hoefler G., Guertl B. (2012). miR-192, miR-194, miR-215, miR-200c and miR-141 are downregulated and their common target ACVR2B is strongly expressed in renal childhood neoplasms. Carcinogenesis.

[40] A Blair S., Kane S.V., Clayburgh D.R., Turner J.R. (2006). Epithelial myosin light chain kinase expression and activity are upregulated in inflammatory bowel disease. Lab. Investig..

[41] E Axelrad J., Lichtiger S., Yajnik V. (2016). Inflammatory bowel disease and cancer: The role of inflammation, immunosuppression, and cancer treatment. World J. Gastroenterol..

[42] Fazal F., Gu L., Ihnatovych I., Han Y., Hu W., Antic N., Carreira F., Blomquist J., Hope T., Ucker D. (2005). Inhibiting Myosin Light Chain Kinase Induces Apoptosis In Vitro and In Vivo. Mol Cell Biol..

[43] Patergnani S., Marchi S., Rimessi A., Bonora M., Giorgi C., Mehta K.D., Pinton P. (2013). PRKCB/protein kinase C, beta and the mitochondrial axis as key regulators of autophagy. Autophagy.

[44] Shin B.K., Kim C.Y., Jung W.Y., Lee H.J., Kim H.K., Kim A. (2011). Proteomic analysis reveals overexpression of moesin and cytokeratin 17 proteins in colorectal carcinoma. Oncol. Rep..

[45] Wang S., Qiu J., Liu L., Su C., Qi L., Huang C., Chen X., Zhang Y., Ye Y., Ding Y. (2020). CREB5 promotes invasiveness and metastasis in colorectal cancer by directly activating MET. J. Exp. Clin. Cancer Res..

[46] Kumaradevan S., Lee S.Y., Richards S., Lyle C., Zhao Q., Tapan U., Jiangliu Y., Ghumman S., Walker J., Belghasem M. (2018). c-Cbl Expression Correlates with Human Colorectal Cancer Survival and Its Wnt/β-Catenin Suppressor Function Is Regulated by Tyr371 Phosphorylation. Am. J. Pathol..

[47] Melo F.D.S.E., Kurtova A.V., Harnoss J.M., Kljavin N., Hoeck J.D., Hung J., Anderson J.E., Storm E.E., Modrusan Z., Koeppen J. (2017). A distinct role for Lgr5+ stem cells in primary and metastatic colon cancer. Nat. Cell Biol..

[48] Barker N., Van Es J.H., Kuipers J., Kujala P., Born M.V.D., Cozijnsen M., Haegebarth A., Korving J., Begthel H., Peters P.J. (2007). Identification of stem cells in small intestine and colon by marker gene Lgr5. Nat. Cell Biol..

[49] Ullmann P., Nurmik M., Schmitz M., Rodriguez F., Weiler J., Qureshi-Baig K., Felten P., Nazarov P.V., Nicot N., Zuegel N. (2019). Tumor suppressor miR-215 counteracts hypoxia-induced colon cancer stem cell activity. Cancer Lett..

[50] (2014). Babraham Bioinformatics. FastQC version 0.11.2. http://www.bioinformatics.babraham.ac.uk/projects/fastqc/.

[51] Bolger A.M., Lohse M., Usadel B. (2014). Trimmomatic: A flexible trimmer for Illumina sequence data. Bioinformatics.

[52] Dobin A., Davis C.A., Schlesinger F., Drenkow J., Zaleski C., Jha S., Batut P., Chaisson M., Gingeras T.R. (2013). STAR: Ultrafast universal RNA-seq aligner. Bioinformatics.

[53] Okonechnikov K., Conesa A., García-Alcalde F. (2016). Qualimap 2: Advanced multi-sample quality control for high-throughput sequencing data. Bioinformatics.

[54] Wingett S.W., Andrews S. (2018). FastQ Screen: A tool for multi-genome mapping and quality control. F1000Research.

[55] Li B., Dewey C.N. (2011). RSEM: Accurate transcript quantification from RNA-Seq data with or without a reference genome. BMC Bioinform..

[56] Love M.I., Huber W., Anders S. (2014). Moderated estimation of fold change and dispersion for RNA-seq data with DESeq2. Genome Biol..

[57] Yu G., Wang L.-G., Han Y., He Q.-Y. (2012). ClusterProfiler: An R Package for Comparing Biological Themes Among Gene Clusters. OMICS A J. Integr. Biol..

[58] Kanehisa M. (2000). KEGG: Kyoto Encyclopedia of Genes and Genomes. Nucleic Acids Res..

[59] Ashburner M., Ball C.A., Blake J.A., Botstein D., Butler H., Cherry J.M., Davis A.P., Dolinski K., Dwight S.S., Eppig J.T. (2000). Gene Ontology: Tool for the unification of biology. Nat. Genet..

[60] (2019). The Gene Ontology Resource: 20 years and still Going strong. Nucleic Acids Res..

