# Supplementary material for: MiR-215-5p Reduces Liver Metastasis in an Experimental Model of Colorectal Cancer through Regulation of ECM-Receptor Interactions and Focal Adhesion
Source: Cancers (Basel). 2020 Nov 26;12(12):3518. doi: 10.3390/cancers12123518 (PMC7760708; doi:10.3390/cancers12123518)
Supplement: Supplementary file 1 [file cancers-12-03518-s001.zip › supplementary file 5.docx]

**Additional file 5:** A list of mRNAs significantly deregulated in HCT-15 cells overexpressing miR-215-5p and associated with focal adhesion based on the KEGG pathway analysis.

| **Ensembl Gene ID** | **Base Mean** | **Log_2_ Fold Change (LFC)** | **LFC SE** | **p-value** | **p-adjusted** | **Gene Name** |
| --- | --- | --- | --- | --- | --- | --- |
| ENSG00000091409 | 6594.938 | -4.31895 | 0.11433 | 0 | 0 | ITGA6 |
| ENSG00000177606 | 9328.405 | 5.14695 | 0.08235 | 0 | 0 | JUN |
| ENSG00000196878 | 24995.39 | 3.30412 | 0.09010 | 2.1E-295 | 9.4E-293 | LAMB3 |
| ENSG00000110092 | 11120.44 | -2.47233 | 0.07868 | 1.6E-217 | 3.7E-215 | CCND1 |
| ENSG00000160691 | 6225.797 | 2.21555 | 0.08084 | 4.5E-166 | 6.1E-164 | SHC1 |
| ENSG00000150093 | 34216.16 | 2.27944 | 0.08480 | 6.9E-160 | 8.5E-158 | ITGB1 |
| ENSG00000130702 | 4798.667 | -2.38222 | 0.09964 | 4.8E-127 | 4E-125 | LAMA5 |
| ENSG00000101335 | 799.3215 | 8.20901 | 0.34641 | 3E-124 | 2.4E-122 | MYL9 |
| ENSG00000118971 | 2484.192 | 10.23550 | 0.42811 | 4.7E-124 | 3.8E-122 | CCND2 |
| ENSG00000162909 | 12750.19 | 1.86410 | 0.08372 | 2E-110 | 1.3E-108 | CAPN2 |
| ENSG00000082781 | 3774.923 | -1.86467 | 0.08730 | 7.7E-102 | 4.7E-100 | ITGB5 |
| ENSG00000159840 | 4449.159 | 1.75372 | 0.08735 | 3.2E-90 | 1.66E-88 | ZYX |
| ENSG00000119630 | 318.6163 | 6.87861 | 0.39675 | 3.57E-67 | 1.28E-65 | PGF |
| ENSG00000188677 | 545.2662 | 8.33049 | 0.49237 | 2.35E-63 | 7.75E-62 | PARVB |
| ENSG00000105974 | 551.3243 | -2.50122 | 0.15292 | 5.59E-61 | 1.75E-59 | CAV1 |
| ENSG00000005884 | 6406.238 | -1.52123 | 0.09355 | 4.05E-60 | 1.24E-58 | ITGA3 |
| ENSG00000101680 | 314.2508 | 3.06270 | 0.19009 | 2.78E-59 | 8.35E-58 | LAMA1 |
| ENSG00000138448 | 4333.049 | -1.54177 | 0.10043 | 1.16E-53 | 3.15E-52 | ITGAV |
| ENSG00000041982 | 897.3563 | -2.40552 | 0.15925 | 2.25E-52 | 5.9E-51 | TNC |
| ENSG00000142156 | 245.9948 | 3.41361 | 0.23319 | 1.02E-49 | 2.53E-48 | COL6A1 |
| ENSG00000169398 | 5670.717 | 1.36152 | 0.09276 | 3.59E-49 | 8.77E-48 | PTK2 |
| ENSG00000161638 | 3467.094 | 2.13581 | 0.14737 | 2.39E-48 | 5.74E-47 | ITGA5 |
| ENSG00000160293 | 1257.401 | 1.51697 | 0.10650 | 1.68E-46 | 3.87E-45 | VAV2 |
| ENSG00000169750 | 763.433 | 2.28625 | 0.16348 | 3.22E-45 | 7.15E-44 | RAC3 |
| ENSG00000050820 | 2239.316 | -1.18015 | 0.08626 | 5.79E-43 | 1.21E-41 | BCAR1 |
| ENSG00000171791 | 173.8928 | 3.25630 | 0.24089 | 1.18E-42 | 2.44E-41 | BCL2 |
| ENSG00000035403 | 7199.996 | -1.73417 | 0.13047 | 7.11E-41 | 1.39E-39 | VCL |
| ENSG00000072110 | 6925.803 | 1.46583 | 0.11067 | 1.56E-40 | 3.02E-39 | ACTN1 |
| ENSG00000019991 | 4984.038 | 17.2965 | 3.70145 | 1.51E-39 | 2.83E-38 | HGF |
| ENSG00000173511 | 913.7936 | 2.07778 | 0.16034 | 3.97E-39 | 7.35E-38 | VEGFB |
| ENSG00000135424 | 155.7821 | 2.97926 | 0.24036 | 2.92E-36 | 4.96E-35 | ITGA7 |
| ENSG00000146648 | 2746.157 | -1.56214 | 0.12482 | 3.05E-36 | 5.17E-35 | EGFR |
| ENSG00000100852 | 4564.57 | -1.54858 | 0.12505 | 1.07E-35 | 1.78E-34 | ARHGAP5 |
| ENSG00000174775 | 996.4484 | -1.66268 | 0.13587 | 5.91E-35 | 9.61E-34 | HRAS |
| ENSG00000213949 | 228.516 | -2.28901 | 0.19054 | 5.59E-34 | 8.78E-33 | ITGA1 |
| ENSG00000127314 | 4481.381 | 1.01593 | 0.08451 | 1.57E-33 | 2.41E-32 | RAP1B |
| ENSG00000129946 | 96.42686 | 4.89341 | 0.40977 | 1.88E-33 | 2.88E-32 | SHC2 |
| ENSG00000132470 | 2229.62 | -12.96054 | 1.31950 | 6.84E-30 | 9.16E-29 | ITGB4 |
| ENSG00000100485 | 644.8871 | 1.66999 | 0.15157 | 8.61E-29 | 1.11E-27 | SOS2 |
| ENSG00000134318 | 3832.685 | -1.11105 | 0.10236 | 1.01E-27 | 1.27E-26 | ROCK2 |
| ENSG00000107263 | 1699.295 | 1.44351 | 0.13642 | 1.32E-26 | 1.56E-25 | RAPGEF1 |
| ENSG00000107242 | 83.44627 | -3.48653 | 0.33850 | 1.17E-25 | 1.33E-24 | PIP5K1B |
| ENSG00000100311 | 272.6086 | -6.83227 | 0.68189 | 1.61E-25 | 1.82E-24 | PDGFB |
| ENSG00000160007 | 3892.869 | -1.30163 | 0.12587 | 2.05E-25 | 2.3E-24 | ARHGAP35 |
| ENSG00000197122 | 1257.625 | 1.34051 | 0.12988 | 2.29E-25 | 2.56E-24 | SRC |
| ENSG00000171608 | 209.3659 | 1.95285 | 0.19161 | 4.05E-25 | 4.47E-24 | PIK3CD |
| ENSG00000197702 | 1222.786 | -1.01368 | 0.10282 | 3.66E-23 | 3.73E-22 | PARVA |
| ENSG00000053747 | 794.9959 | -11.35502 | 1.30618 | 1.95E-22 | 1.91E-21 | LAMA3 |
| ENSG00000058335 | 116.5374 | -7.14152 | 0.73171 | 3.23E-22 | 3.13E-21 | RASGRF1 |
| ENSG00000141736 | 2671.037 | -1.12678 | 0.11793 | 6.43E-22 | 6.12E-21 | ERBB2 |
| ENSG00000065534 | 79.0599 | -4.77378 | 0.51993 | 6.65E-22 | 6.31E-21 | MYLK |
| ENSG00000215375 | 123.9217 | -2.14768 | 0.24050 | 8.96E-20 | 7.55E-19 | MYL5 |
| ENSG00000134871 | 158.092 | -11.24766 | 2.98479 | 1.6E-17 | 1.19E-16 | COL4A2 |
| ENSG00000117020 | 255.2703 | 9.75544 | 1.31153 | 2.34E-17 | 1.71E-16 | AKT3 |
| ENSG00000187498 | 171.624 | -11.13136 | 2.97759 | 3.61E-17 | 2.63E-16 | COL4A1 |
| ENSG00000171914 | 349.2203 | -1.31934 | 0.16031 | 7.89E-17 | 5.62E-16 | TLN2 |
| ENSG00000115414 | 169.1799 | -9.61887 | 1.30078 | 1.22E-16 | 8.55E-16 | FN1 |
| ENSG00000112715 | 2260.872 | -1.02982 | 0.12553 | 1.29E-16 | 9.08E-16 | VEGFA |
| ENSG00000115221 | 127.9864 | -7.42803 | 0.97943 | 2.39E-16 | 1.65E-15 | ITGB6 |
| ENSG00000105855 | 97.23365 | -6.69235 | 0.82459 | 1.34E-15 | 8.84E-15 | ITGB8 |
| ENSG00000196569 | 84.77638 | -10.26711 | 2.89244 | 1.21E-14 | 7.51E-14 | LAMA2 |
| ENSG00000058085 | 491.9706 | -5.99395 | 0.84316 | 1.68E-14 | 1.03E-13 | LAMC2 |
| ENSG00000121879 | 948.6616 | 1.07477 | 0.14204 | 2E-14 | 1.22E-13 | PIK3CA |
| ENSG00000137801 | 1612.621 | -7.61606 | 1.11087 | 1.47E-13 | 8.5E-13 | THBS1 |
| ENSG00000145431 | 67.79375 | -9.79462 | 2.84303 | 1.84E-13 | 1.05E-12 | PDGFC |
| ENSG00000092758 | 190.0951 | 1.28370 | 0.20312 | 1.09E-10 | 5.08E-10 | COL9A3 |

LFC – logarithmic fold-change (miR-215-5p compared to mock), SE – standard error
