# Supplementary material for: MiR-215-5p Reduces Liver Metastasis in an Experimental Model of Colorectal Cancer through Regulation of ECM-Receptor Interactions and Focal Adhesion
Source: Cancers (Basel). 2020 Nov 26;12(12):3518. doi: 10.3390/cancers12123518 (PMC7760708; doi:10.3390/cancers12123518)
Supplement: Supplementary file 1 [file cancers-12-03518-s001.zip › supplementary file 6.docx]

**Additional file 6:** A list of mRNAs significantly deregulated in HCT-15 cells overexpressing miR-215-5p and associated with ECM-receptor interaction based on the KEGG pathway analysis.

| **Ensembl Gene ID** | **Base Mean** | **Log_2_ Fold Change (LFC)** | **LFC SE** | **p-value** | **p-adjusted** | **Gene Name** |
| --- | --- | --- | --- | --- | --- | --- |
| ENSG00000091409 | 6594.94 | -4.31896 | 0.11433 | 0 | 0 | ITGA6 |
| ENSG00000196878 | 24995.4 | 3.30412 | 0.09010 | 2E-295 | 9E-293 | LAMB3 |
| ENSG00000072571 | 1337.45 | -3.41648 | 0.11955 | 2E-180 | 2E-178 | HMMR |
| ENSG00000150093 | 34216.2 | 2.27944 | 0.08481 | 7E-160 | 9E-158 | ITGB1 |
| ENSG00000188157 | 9822.93 | -2.14475 | 0.08604 | 9E-138 | 8E-136 | AGRN |
| ENSG00000130702 | 4798.67 | -2.38223 | 0.09964 | 5E-127 | 4E-125 | LAMA5 |
| ENSG00000082781 | 3774.92 | -1.86467 | 0.08731 | 8E-102 | 5E-100 | ITGB5 |
| ENSG00000005884 | 6406.24 | -1.52123 | 0.09355 | 4E-60 | 1.2E-58 | ITGA3 |
| ENSG00000101680 | 314.251 | 3.06270 | 0.19009 | 2.8E-59 | 8.3E-58 | LAMA1 |
| ENSG00000138448 | 4333.05 | -1.54178 | 0.10043 | 1.2E-53 | 3.1E-52 | ITGAV |
| ENSG00000041982 | 897.356 | -2.40553 | 0.15926 | 2.2E-52 | 5.9E-51 | TNC |
| ENSG00000142156 | 245.995 | 3.41361 | 0.23319 | 1E-49 | 2.5E-48 | COL6A1 |
| ENSG00000161638 | 3467.09 | 2.13581 | 0.14738 | 2.4E-48 | 5.7E-47 | ITGA5 |
| ENSG00000173402 | 2268.64 | -1.36328 | 0.10747 | 2.9E-37 | 5.1E-36 | DAG1 |
| ENSG00000135424 | 155.782 | 2.97926 | 0.24037 | 2.9E-36 | 5E-35 | ITGA7 |
| ENSG00000213949 | 228.516 | -2.28902 | 0.19055 | 5.6E-34 | 8.8E-33 | ITGA1 |
| ENSG00000132470 | 2229.62 | -12.96050 | 1.31950 | 6.8E-30 | 9.2E-29 | ITGB4 |
| ENSG00000053747 | 794.996 | -11.35500 | 1.30618 | 2E-22 | 1.9E-21 | LAMA3 |
| ENSG00000134871 | 158.092 | -11.24770 | 2.98479 | 1.6E-17 | 1.2E-16 | COL4A2 |
| ENSG00000187498 | 171.624 | -11.13140 | 2.97759 | 3.6E-17 | 2.6E-16 | COL4A1 |
| ENSG00000115414 | 169.18 | -9.61888 | 1.30079 | 1.2E-16 | 8.5E-16 | FN1 |
| ENSG00000115221 | 127.986 | -7.42803 | 0.97943 | 2.4E-16 | 1.7E-15 | ITGB6 |
| ENSG00000105855 | 97.2337 | -6.69236 | 0.82459 | 1.3E-15 | 8.8E-15 | ITGB8 |
| ENSG00000168743 | 177.63 | -9.04633 | 1.29270 | 4.5E-15 | 2.9E-14 | NPNT |
| ENSG00000138759 | 1238.43 | -1.12977 | 0.14776 | 1.1E-14 | 6.6E-14 | FRAS1 |
| ENSG00000196569 | 84.7764 | -10.26710 | 2.89244 | 1.2E-14 | 7.5E-14 | LAMA2 |
| ENSG00000058085 | 491.971 | -5.99396 | 0.84317 | 1.7E-14 | 1E-13 | LAMC2 |
| ENSG00000142798 | 2100.33 | -2.45741 | 0.34115 | 9E-14 | 5.2E-13 | HSPG2 |
| ENSG00000137801 | 1612.62 | -7.61606 | 1.11087 | 1.5E-13 | 8.5E-13 | THBS1 |
| ENSG00000092758 | 190.095 | 1.28370 | 0.20312 | 1.1E-10 | 5.1E-10 | COL9A3 |

LFC – logarithmic fold-change (miR-215-5p compared to mock), SE – standard error
