# Supplementary figures and images for: MiR-215-5p Reduces Liver Metastasis in an Experimental Model of Colorectal Cancer through Regulation of ECM-Receptor Interactions and Focal Adhesion
Source: Cancers (Basel). 2020 Nov 26;12(12):3518. doi: 10.3390/cancers12123518 (PMC7760708; doi:10.3390/cancers12123518)

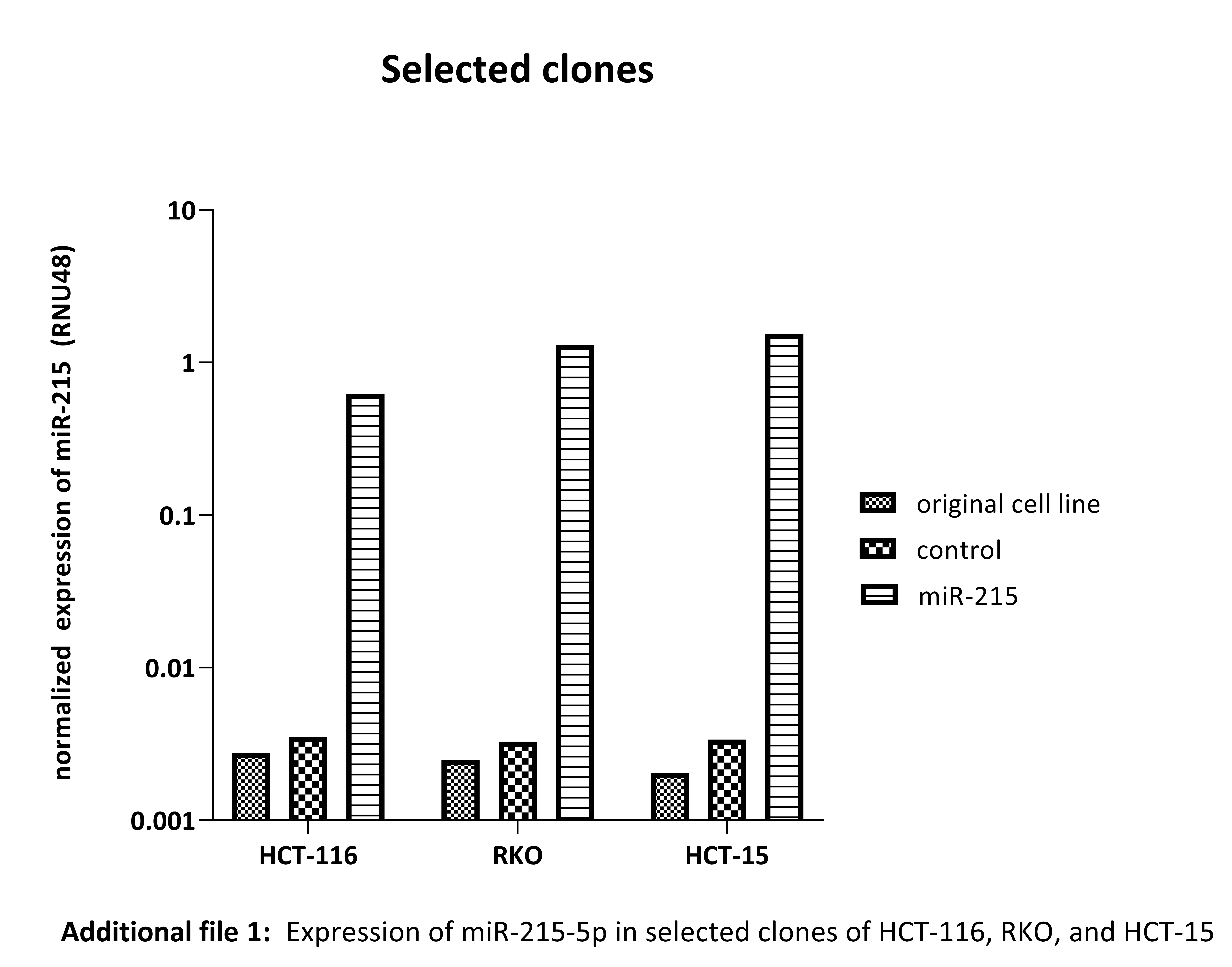

Supplement: Supplementary file 1 [file cancers-12-03518-s001.zip › supplementary File 1.png]

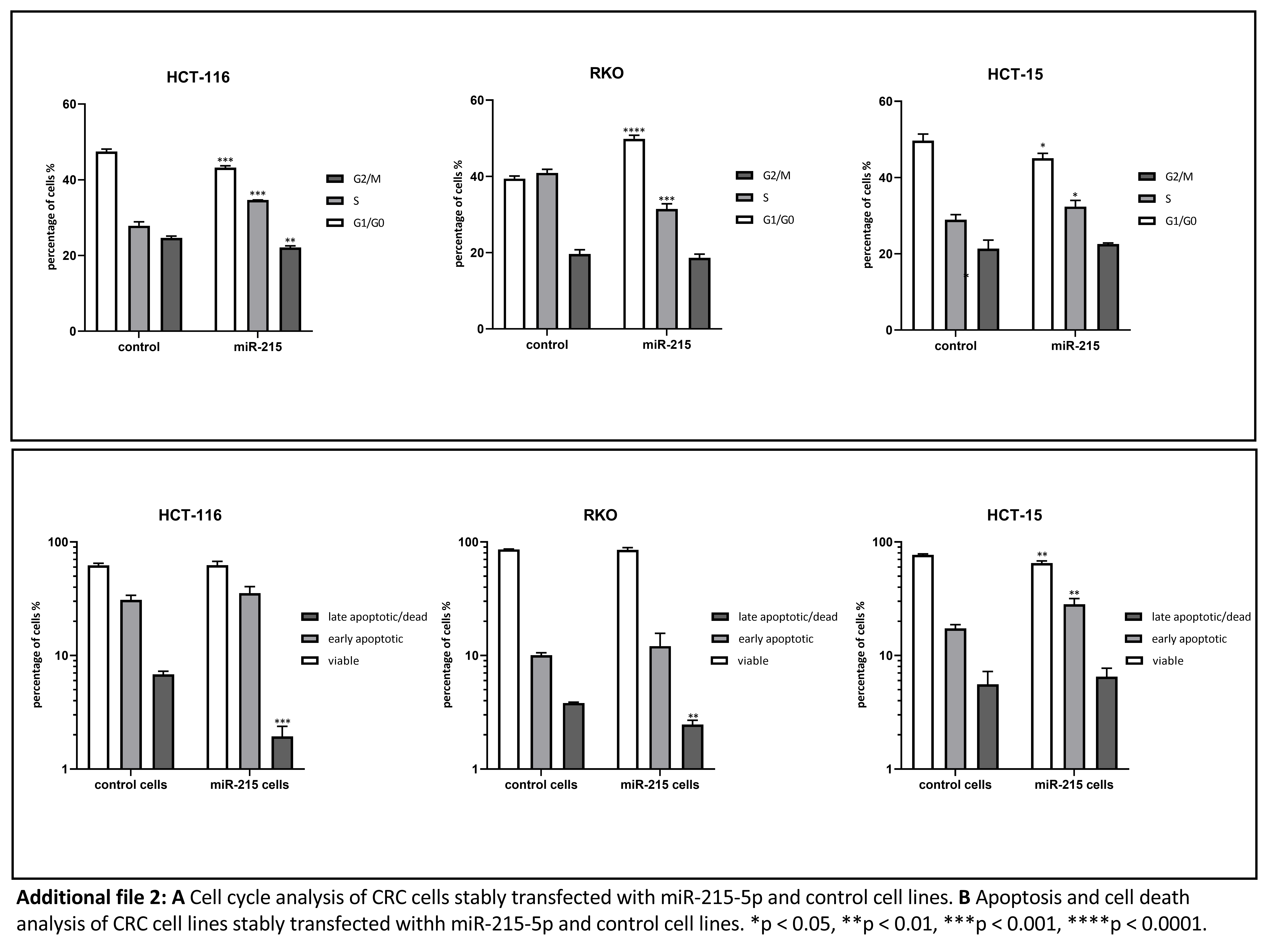

Supplement: Supplementary file 1 [file cancers-12-03518-s001.zip › supplementary file 2.png]
